# Analysis of indoxyl sulfate in biological fluids with emphasis on sample preparation techniques: A comprehensive analytical review

**DOI:** 10.1016/j.heliyon.2024.e35032

**Published:** 2024-07-23

**Authors:** Samira Shafiee, Siavoush Dastmalchi, Afshin Gharekhani, Ali Shayanfar

**Affiliations:** a Student Research Committee, Tabriz University of Medical Sciences, Tabriz, Iran; b Biotechnology Research Center, Tabriz University of Medical Sciences, Tabriz, Iran; c Faculty of Pharmacy, Tabriz University of Medical Sciences, Tabriz, Iran; d Pharmaceutical Analysis Research Center, Tabriz University of Medical Sciences, Tabriz, Iran

**Keywords:** Bioanalysis, Chronic kidney disease (CKD), Indoxyl sulfate (IS), Sample preparation, Uremic toxin

## Abstract

The uremic toxin indoxyl sulfate (IS) has been related to the development of various medical conditions notably chronic kidney disease (CKD). Hence, quantification of this biomarker in biological fluids may be a diagnostic tool to evaluate renal system functionality. Numerous analytical methods including liquid chromatography, gas chromatography, spectroscopy, and electrochemical techniques have since been used to analyze IS in different biological fluids. The current review highlights the relevant studies that assessed IS with a special focus on sample preparation, which is essential to reduce or eliminate the effect of endogenous components from the matrix in bioanalysis.

## 1 Introduction

Many different compounds are eliminated from the human system through healthy kidneys [1]. Uremic syndrome, which is potentially life-threatening when left untreated in its latter stages, occurs by failure in kidney clearance capacity [2]. The elimination pattern of uremic toxins by dialysis is generally classified into three categories: microscopic water-soluble substances (including urea), protein-bound compounds (including indoles and phenols), and middle substances (including b2-microglobulin) [2]. Studies have shown these compounds have an impact on the progression of chronic kidney disease (CKD), however, some reports also imply that small water-soluble agents may play a role as well [2,3]. It is obvious that the protein-bound solutes have limited clearance due to the availability of only the unbound fraction for distribution via the membrane in dialysis systems [4].

The most frequently utilized marker compounds for monitoring the level of uremic retention of the protein-bound solutes in hemodialysis are indoxyl sulfate (IS) and p-cresyl sulfate. These biomarkers are of great importance since it is believed that they play important roles in the development of uremic syndrome. IS, a metabolite of dietary l-tryptophan, is thought to be a crucial factor in CKD [5]. According to several investigations, the accumulation of protein-bound uremic toxins like IS in blood adversely impacts a wide variety of biological processes, because they may lead to adverse reactions in kidneys, heart, and bones [[6], [7], [8], [9], [10]]. For example, renal damage caused by increased serum levels of IS following an acute myocardial infarction (AMI) was well documented [11]. Therefore, when evaluating kidney disease, assessing the IS concentration and other uremic toxins may act as a reliable biomarker for starting the immediate treatments required to maintain kidney function [12]. The concentration of IS in healthy people and early stage of CKD patients was reported less than 10 μg/mL and in CKD patients is in the range of 10–30 μg/mL. This value for end-stage renal disease (ESRD) of CKD patients more than 40 μg/mL has been reported [13,14]. The molecular weight (213.21 g/mol) and the protein-binding characteristics (more than 90 %) and the necessity of its analysis in biological samples make the detection of IS difficult. Therefore, liquid chromatography (LC) with mass or fluorescence detectors with a sample preparation technique are inevitable.

There are various review articles in the literature which discussed the clinical roles of IS as a biomarker in CKD [15,16] and cardiovascular diseases [17,18]. However, there is no review about the development of analytical techniques for monitoring IS. The current review discusses different analytical techniques that have been used for quantifying IS in various biological fluids, with emphasis on sample preparation and validation parameters.

Trying to find the most frequently used separation methods in clinical investigations, we explored and considered the related information in several clinical articles from 1986 to 2023. The bibliometric analysis was conducted by searching techniques in Scopus and Google Scholar using keywords like IS, determination, quantification, plasma, urine, and biological sample.

## 2 Analytical techniques for quantification of IS

Numerous separation methods, particularly those based on chromatography, such as gas chromatography (GC) and liquid chromatography (LC) have been applied for assessing different uremic toxins, especially IS. However, some non-chromatographic methods such as spectroscopy and electrochemical methods were developed in the literature.

### 2.1 Liquid chromatography

**Fig. 1:**
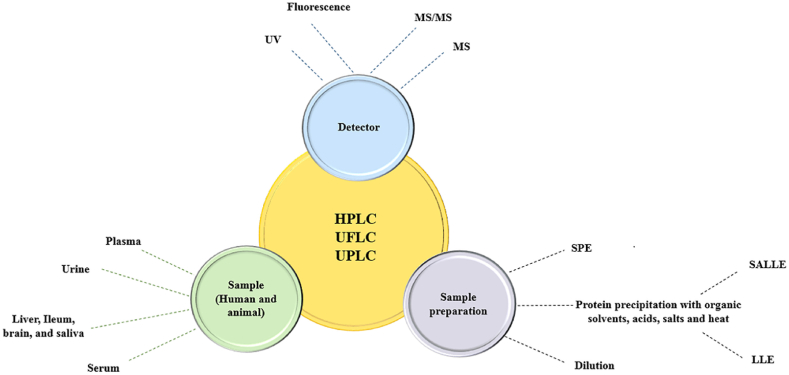
A schematic figure of available Liquid Chromatography techniques for determination of IS in biofluids.

Generally, chromatography methods is common methods for analysis of drugs and biomarkers in biological samples because of its capability in separation of analytes from the matrix i.e., plasma and urine. Due to the low volatility of uremic toxins, LC is the most used separation method for determining the concentration of uremic compounds in the biological matrix [3]. Table 1 listed a number of available LC techniques that have been developed to analyze IS in analytical determinations. Fig. 1 also illustrated a schematic of the mentioned techniques in Table 1. The linear range of methods depend on several variables, including the sensitivity of the chosen instrumental method of analysis, and type of sample treatment technique [[19], [20], [21]].

**Table 1:** List of reported liquid chromatography method for quantification of IS in biological samples.

No	Analysis Method	Detector	Linear range	Sample matrices	Sample preparation	Ref.
1	HPLC	MS/MS	0.1–100 μg/mL	Human and animals plasma	Protein precipitation with ACN	[22]
2	HPLC	MS/MS	500–10000 ng/mL	Human serum	Protein precipitation with ACN	[23]
3	HPLC	MS/MS	0.5–500 μM	Human urine	Diluted with formic acid	[24]
4	HPLC	MS/MS	1.5–200 ng/mL	Human saliva	Protein precipitation with ACN	[25]
5	HPLC	MS/MS	1.9–194.6 μM	Human serum	Protein precipitation with formic acid and ACN	[26]
6	HPLC	MS-MS	0.2–349 mM	Human serum	Protein precipitation with ACN	[27]
7	HPLC	MS/MS	1–50000 ng/mL	Human serum	Protein precipitation with methanol	[28]
8	HPLC	MS/MS	5–40 μM	Human serum, urine, and cell culture	Dilution with formic acid and water	[29]
9	HPLC	MS/MS	0.01–10 μg/mL	Human plasma	Protein precipitation with ice-cold ACN and formic acid	[30]
10	HPLC	MS/MS	Total serum concentration:0.002–10 mg/dL Free-form concentration:0.0005–1 mg/dL	Human serum	Protein precipitation with ACN	[31]
11	HPLC	MS/MS	0.1–20 μM	Human serum and gastric juice	Protein precipitation with formic acid and ACN	[32]
12	UPLC	MS/MS	0.019–9.860 μg/mL	Human urine, plasma, and serum	Protein precipitation with methanol and ACN (1:1)	[33]
13	HPLC	MS	0.1–500 μM	Human serum	Dilution with formic acid and SPE with commercial cartridge	[34]
14	UPLC	MS/MS	156.250–20000.000 ng/mL	Human serum	Protein precipitation with ACN	[35]
15	UPLC	MS/MS	0.2–80 μg/mL	Human serum	Protein precipitation with ACN	[36]
16	UPLC	MS/MS	0.019–9.860 μg/mL	Human urine, plasma, and serum	Protein precipitation with methanol and ACN (1:1)	[33]
17	UPLC	MS/MS	0.05–200 μg/mL	Human plasma	SPE	[37]
18	UPLC	MS/MS	0.05–5.18 mg/L	Human serum	Protein precipitation with ACN	[12]
19	UPLC	MS/MS	0.1–40 μg/mL	Human serum	Denaturation of protein by heat	[38]
20	UPLC	MS	480-48 000 ng/mL	Human urine	Dilution with water	[39]
21	UFLC	MS/MS	1000–2000 × 10^4^ ng/mL	Human serum and urine	Protein precipitation with ACN and methanol	[40]
22	HPLC	Fluorescence	0.5–10 μg/mL	Human plasma	Protein precipitation with ethanol and SALLE with NaCl	[41]
23	HPLC	Fluorescence	2.5–50 mM	Human plasma	Protein precipitation with ACN	[42]
24	HPLC	Fluorescence	0–1500 μM	Human serum	Protein precipitation with ethanol	[43]
25	HPLC	Fluorescence	1.6 and 400.0 μM	Human serum	Protein precipitation with cold acetone and LLE with dichloromethane	[44]
26	HPLC	Fluorescence	–	Human serum	Protein precipitation with ACN and LLE with dichloromethane	[45]
27	HPLC	Fluorescence	0.5–80.0 μg/mL	Human serum	Protein precipitation by ACN	[46]
28	UPLC	Fluorescence	0.1–100 μM	Human serum	Protein precipitation with ethanol and LLE with dichloromethane	[47]
29	UPLC	Fluorescence	0.01–20 μg/mL	Human urine	Dilution with methanol and water	[48]
30	HPLC	UV	50-25 000 pM	Human plasma	Protein precipitation with ACN	[49]
31	HPLC	UV	0.01–0.15 mg/mL	Human plasma	SPE (carbon nanotubes)	[50]

HPLC: High Performance Liquid Chromatography, UPLC: Ultra Performance Liquid Chromatography, UFLC: Ultra-Fast Liquid Chromatography, ACN: Acetonitrile, LLE: Liquid-liquid extraction, SALLE: Salting-out assisted liquid liquid extraction, SPE: Solid phase extraction, TCA: Trichloroacetic acid.

#### 2.1.1 LC methods with mass detector

The liquid chromatography followed by mass (MS) or tandem mass spectrometry detector (MS/MS) was the mostly utilized approach in clinical research to identify uremic toxins, mainly IS, which is the main emphasis of the current study. Generally, mass detectors are more sensitive detection techniques in compared with UV detector.

The aims of most clinical studies were to investigate the relationship between serum IS concentration and different clinical implications such as kidney injury [11], endothelial function in non-dialysis CKD patients [51], the renal function [52], all-cause mortality [53], aortic arterial stiffness [54], iron dynamics index in patients with end-stage kidney disease (ESKD) [55], vascular disease in CKD patients [56], Nrf2 expression through activation of NF-κB [57], cardiovascular disease [17,[58], [59], [60]], immune system [61], bone histomorphometry in pre-dialysis CKD patients [62], anemia-related CKD [[63], [64], [65]], severity of coronary atherosclerosis [66,67], bone abnormalities linked to fibroblast growth factor-23 (FGF-23) in humans with CKD [68], cardiovascular phenotype in children with CKD [69], sarcopenia and malnutrition in elderly patients with advanced CKD [70], skeletal resistance in hemodialysis patients [71], postangioplasty thrombosis of dialysis grafts [72], arotid-femoral pulse wave velocity in patients with CKD [73]. Without doubt, in analytical laboratories, the LC-MS technique has received considerable attention. In recent years, several scientific groups have developed the LC-MS/MS methods for the quantification of IS [22,[24], [25], [26], [27], [28],^32^,[74], [75], [76], [77], [78], [79]].

Chromatography-based approaches are time- and labor-intensive, and the associated specialized equipment for analysis is expensive. Due to these drawbacks, the gold-standard LC-MS and LC-MS/MS approaches are not compatible with in real-time on-site and routine clinical studies [80,81].

The most important and challenging phase of an analysis is frequently sample preparation since it takes the most time to get the target analyte out of the matrix. Each matrix also presents its own special difficulties [82]. For instance, whole blood contains red blood cells that typically need to be lysed [82], plasma is comprised of dissolved materials, predominantly proteins such as albumin and globulin, coagulants, electrolytes, immunoglobulins [83] and also urine has a high salt content, urea, creatinine, uric acid, chloride, sodium, potassium, sulfate, ammonium, phosphate, and other ions and molecules in lower amounts [84]. The appropriate extraction strategy is frequently determined by the distinctive properties of each analyte and matrix [82,85].

Stanfel et al. [49] used an ion-pairing reversed-phase liquid-chromatographic method by tetrabutylammonium phosphate as an ion-pairing agent for the assay of IS in plasma from normal subjects and patients with various degrees of renal failure.

Moreover, numerous ultra-high performance liquid chromatography-tandem mass spectrometry (UPLC-MS/MS) techniques have been presented for assessing IS either individually or in combination with other analytes. Boelaert et al. [38] presented a novel UPLC-MS/MS technique for simultaneous analysis of seven uremic retention toxins including IS with cardiovascular implication in CKD patients. The method included separation on a C18 column was followed by negative electrospray ionization and tandem mass spectrometric detection. All seven solutes could only be detected in the negative mode, which was therefore selected. Mass spectral ionization, fragmentation, and acquisition conditions were optimized on the tandem quadrupole mass spectrometer by using electrospray ionization (ESI).

##### 2.1.1.1 Sample preparation for LC methods with mass detector

Investigation of the reported analytical method for quantification of IS using UPLC and HPLC with MS or MS/MS indicate that because of high sensitivity and separation capability of these methods, dilution and protein precipitation are commonly used. In order to prepare samples for instrumental analysis, dilution is also one of the required sample pretreatment techniques. However, it is impossible to eliminate impurities and undesirable analytes by dilution. Usually, mobile phase is chosen as the diluent before performing chromatographic analysis since the productivity of dilution depends on the diluents and their fractions that are chosen [3]. Using dilution technique, Baird et al. [24] established a rapid method for measuring tryptophan, indole, and IS in urine samples that involved diluting urine samples with formic acid before injecting them onto LC-MS/MS for analysis.

Protein precipitation is reasonably easy to apply and offers rapid sample cleanup, particularly for blood, plasma, and serum samples. The methodology is centered on applying an organic solvent, acid, or salt to the sample under consideration [86]. The organic solvents, e.g., acetone, methanol, ethanol and acetonitrile (ACN), are capable of separating nearly 95 % of plasma proteins. Common solvent in bioanalysis is ACN which has a stronger precipitability than other solvents. Acids (perchloric acid, trichloroacetic acid, or phosphoric acid) are also excellent precipitation agents [87]. Researchers used different protein precipitation methods to prepare samples suitable for LC-MS/MS analysis. For the determination of the uremic toxins like IS, ACN was used by some groups [22,25,74,78] to precipitate proteins [3], while others (Fabresse et al. [28] and Zeng et al. [79]) used methanol to precipitate the proteins. Kanemitsu et al. [26] and Choi et al. [32] used 0.1 % formic acid in ACN for protein precipitation. In the developed UPLC-MS/MS method, protein precipitation by ACN was the most employed sample preparation technique [12,35,36,88] for purifying human biofluids for the detection of the target analytes. These agents usually are applied for protein precipitation and sample preparation in chromatographic methods. However, it is not a powerful method for complete clean-up of plasma sample. In few cases where IS was analyzed using LC-mass or LC method linked to other detector methods, the extraction step was applied for sample preparation to clean-up and preconcentrate the analyte. Oda et al. [37] has used UHPLC-MS/MS system comprising a Nexera X2 LC system and a triple quadrupole mass spectrometer and commercial solid-phase extraction (SPE) method to prepare the samples. It is worth mentioning that in order to measure analytes in biological samples like plasma and urine, SPE are frequently utilized in sample preparation procedures. With the use of this method, the analytes can be preconcentrated while biological matrix elements can be eliminated [89]. Standard methods for biological sample preparation, such as liquid-liquid extraction (LLE), are quick, easy and simple procedures that do not call for particular instruments [90]. A significant amount of biological substances and organic solvents are required for the traditional LLE. In order to obtain a pre-concentrated sample that is suitable for injection into the chromatographic system, the organic solvent must be removed from the sample and the extracted analyte should be reconstitute in an appropriate solvent [90]. Furthermore, LLE is frequently combined with protein precipitation. Combining several extraction methods makes it possible to produce cleaner extracts and increases extraction effectiveness. Different non-water miscible solvents or solvent mixes have been used when LLE is linked to protein precipitation, including isopropyl ether, ethyl acetate, dichloromethane, diethyl ether, hexane, and dichloromethane [3].

#### 2.1.2 LC methods with fluorescence detector

Fluorescence detector in comparison with UV and MS detectors has a number of important benefits such as high sensitivity and selectivity, low background noise and the ability to detect low levels of fluorescent compounds. However, its disadvantages are compatibility issues with some solvents and columns. Some compounds such as IS only has native fluorescence. Therefore, fluorescence detector could use for the analysis of IS in biological samples.

De Loor et al. [44], announced an LC method with fluorescence detector to determine IS in uremic and normal serum. Considering LLE as a type of extraction, which is frequently combined with protein precipitation, they applied LLE with dichloromethane after protein precipitation with cold acetone. Clearly, combining various extraction methods causes a cleaner extract and increased the extraction yield [3].

In order to study reference ranges and biological variation in healthy individuals, Pretorius et al. [47] devised a UPLC approach with fluorescence detection. They provided details of a reliable analytical technique that measures both IS and p-cresyl sulfate (another uremic toxic) and has a short chromatography time. Clinical investigations that examine these uremic toxins must take into account the information on reference ranges and intra-individual variations. Sample preparation was carried out under deproteinization with ethanol and LLE with dichloromethane.

HPLC method using fluorescence detector was also reported by Silva et al. [41] for the analysis of IS. A reversed-phase monolithic column was employed for chromatographic separation. In order to precipitate proteins, human plasma standards and samples containing uremic toxins were added to ethanol containing eugenol as the internal standard. NaCl was subsequently added to facilitate salting-out aided deproteinization. In accordance with the fundamentals of green analytical chemistry, sample preparation was effortless and only required a small amount of plasma (50 μL). This aspect enhances the greenness and sustainability of the suggested approach, leading towards the current direction of green analytical development, along with a green extraction solvent (ethanol), a less harmful internal standard (eugenol).

Al Zaabi et al. [42], Cheng et al. [91], and Chun et al. [46] also developed HPLC method with fluorescence detection using ACN to remove the proteins from the matrix. Paats et al. [92] by diluting and filtering the samples established HPLC method coupled with fluorescence detector to determine IS. Calaf et al. [43] established an analytical method based on ion-pairing HPLC-fluorescence method using tetrabutyl ammonium iodide as ion-pairing agent. Zou et al. [45] also described a strategy based on HPLC method with fluorescence detector promoting deproteinization with ACN and LLE with dichloromethane.

#### 2.1.3 LC methods with UV detector

UV detectors are common and more accessible and economical in compared with mass and fluorescence detectors. Because of its low sensitivity compared with other detectors, rarely used for the analysis of IS in biological samples. AL Othman et al. [50] proposed a comparative and simultaneous determination of IS and sodium butyrate in human plasma using SPE and HPLC techniques with UV detection by two new form of columns, i.e., phenyl and SunShell which can interact with analytes based on hydrogen bonding and π-π interactions. In addition, they applied carbon nanotubes as solid phase for the extraction of the target analytes.

### 2.2 Gas chromatography

For several decades, GC has been used to analyze biological fluids [93]. While LC has been widely employed to analyze of compounds with diverse physicochemical properties, GC has been utilized for the identification and quantification of volatile and semi-volatile compounds. Because of this volatility, GC is much quicker than LC methods. The disadvantages of GC are due to its application on volatile compounds. Non-volatile compounds such as IS need to be derivatized to analyze them using GC [94,95]. Some of the advantages of GC coupled with MS over LC/MS include greater peak capacity, chromatographic resolution and a single mobile phase. These benefits hold particular significance when examining complex matrices.

Metabolomics analysis is an appropriate example for analysis of IS using GC [96]. Concerning this, Omori et al. identified novel biomarker candidates including inositol and IS for atherosclerosis in Japanese patients with type 2 diabetes (T2DM) using GC/MS-based non-targeted metabolomics [97]. The proposed GC/MS based analytical technique involved a two-step derivatization procedure; first derivatization with methoxyamine hydrochloride and then derivatizing with N-methyl-N-trimethylsilyl-trifluoroacetamide using plasma of patients with T2DM. Three preparation methods i.e., methanol: water: chloroform (5:1:2), water: ACN (1:4) and water: methanol (1:4) were applied for the clean-up of plasma samples and the best method was selected based on protein residual ratio. The water: ACN (1:4) with protein residual ratio equal with 0.5 % was ultimately selected as clean-up technique.

### 2.3 Non-chromatographic methods

Chromatographic methods are common analytical techniques for quantifying drugs and metabolites in biological samples. However, these methods are time-consuming and expensive techniques for routine analysis. Therefore, electrochemical spectroscopy and other methods were also developed to analyze IS in biological samples.

In the analysis of real samples, removing the matrix of plasma or urine is a challenge, and variation in the biological sample of each person is a critical point. This is more crucial for IS because similar structure to amino acids, especially with tryptophan. In addition, analysis of IS is necessary for CKD patients, and generally, several medicines are administrated for them which should be considered in the analysis of IS. Therefore, interfering substances in biological matrixes are a common limitation for obtaining accurate data for IS and developing these methods for routine analysis.

#### 2.3.1 Electrochemical methods

**Fig. 2:**
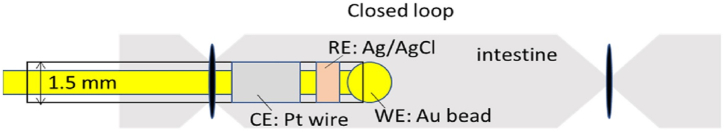
An illustration of the closed-loop rat intestine all-in-one electrode system for IS detection. Reproduced with permission from ref 94. Copyright (2022) Elsevier.

Electrochemical methods were which have simplicity, low cost and speed in comparison with chromatography techniques [[98], [99], [100], [101], [102]] also applied for the quantification of IS in biological samples. To establish an all-in-one device constructed using the 2-mercaptobenzimidazole (MBI)-modified gold bead electrodes, Fujita et al. [103] tried to observe the secretion of IS in the rat intestinal loop in real-time. This approach is receiving attention for the real-time detection of IS in the intestine, which is among the most significant excretion pathways for medicines and other targets. Thus, the establishment of an analytical technique to evaluate the IS concentration in the intestine is necessary. Fig. 2 illustrated a schematic view of the all-in-one electrode fixed in the closed intestinal loop. It was composed of an MBI monolayer-modified gold bead electrode (WE), Platinum (Pt) wire, and Ag/AgCl wire. The limit of detection of the all-in-one electrode identified to be 0.04 μg/mL. To concluded, they suggested that IS is excreted through the intestine when its level is high and the self-assembled monolayer (SAM)-modified gold bead electrode can be used as an easy and sensitive alternative for identifying IS secreted in the intestine regularly.

To develop square-wave voltammetry as a rapid methodology for the measurement of IS in urine samples, Filik et al. [104] used high-performance disposable screen-printed graphene electrode for sensitive estimation of IS using square-wave voltammetry. The outcomes demonstrated that an ideal platform for the assessment of IS can be provided using the graphene screen printed electrode (GR-SPE). With low limit of detection, wide linear dynamic range, and ideal selectivity, the GR-SPE was effectively applied to detect free IS. In general, electrochemical sensing is capable of detecting the analytes which are important for clinical diagnosis and disease monitoring with rapid and selective methods.

#### 2.3.2 Fluorescence spectroscopy

Fluorescence spectroscopy against chromatography techniques is accessible, simple, and cheap. However, selectivity is the main disadvantage of these techniques for the quantification of biomarkers in bioanalysis.

It is of note that native fluorescence spectroscopy because of native fluorescence of IS was applied for the analysis of IS. Holmar [105] et al. suggested fluorescence spectroscopy as a feasible method to measure the removal ratio (RR) of IS using just the fluorescence outcomes of the tested dialysate, and the obtained parameter can be utilized to represent the clearance of protein-bound uremic toxins during the dialysis procedure.

**Fig. 3:**
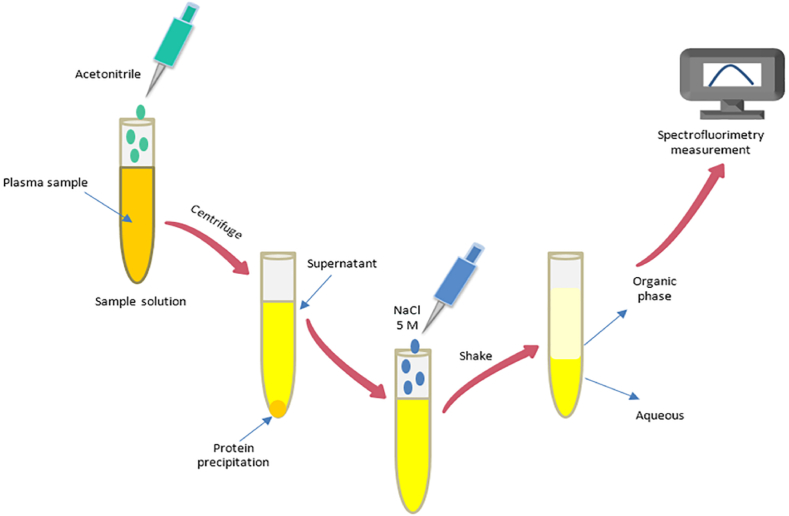
IS extraction by salting-out liquid liquid extraction method and the analysis using spectrofluorimetry.

Norouzi et al. [14] also developed an analytical method based on the fluorescence technique for quantification of IS in the human plasma of patients with CKD after salting-out assisted liquid–liquid extraction (SALLE). Plasma samples were deproteinized by acetonitrile and the clean-up of plasma was performed by salting-out of acetonitrile phase by sodium chloride. The presented method had a linear range between 2.5 and 40 μg/mL and appropriate selectivity for quantification of IS in plasma in the presence of other compounds which prescribed for these patients. The concentration of IS in real plasma samples of CKD patient was in the range of 10–30 μg/mL. Fig. 3 shows a schematic presentation of IS extraction by established extraction method and analysis using spectrofluorimetry.

Rajasekaran et al. [106] aimed at using steady state and time-resolved fluorescence spectroscopy at 280 nm and 350 nm excitation to characterize the urine of both healthy individuals and patients with proven malignancy. It has been found that changed spectral signatures at 280 nm and 350 nm excitation are caused by the metabolites IS and neopterin and its derivatives. Also, statistics were applied to examine the fluorescence emission spectra of urine from both cancer patients and healthy individuals. The findings imply that fluorescence emission spectroscopy may be used to distinguish between urine samples from cancer patients and those from healthy individuals.

In 2018, Caggiano et al. [74] proposed a spectrophotometry method to determine serum IS levels that could be integrated into clinical practice. To determine the IS in hemodialysis patients and healthy volunteers, they used a derivatization reaction to transform the serum IS into indigo blue (an organic compound with a distinctive blue color) to measure the IS using spectrophotometric assay. The proposed method could represent a valid tool for the analysis of gut-derived IS and monitoring dialysis efficacy and CKD progression.

**Fig. 4:**
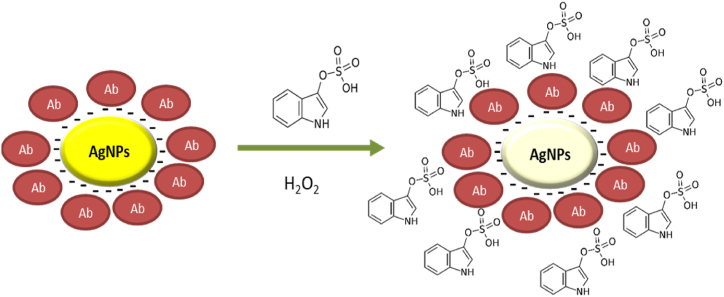
A schematic of colorimetric sensor method based on citrate-capped silver nanoparticles for quantification of indoxyl sulfate (IS). Ab: Albumin.

Schiefer et al. [107] developed a colorimetric sensor based on albumin bound to citrate-capped silver nanoparticles. It was capable of detecting IS and *p*-cresylsulfate. The above mentioned toxins accelerate the hydrogen peroxide-oxidation of citrate-capped silver nanoparticles, which affects the targeted surface plasmon resonance and enables the suggested colorimetric sensing technique (Fig. 4). The approach indicated a linear range for IS and *p*-cresylsulfate concentrations ranging from 15 to 100 mg/L. These aspects of the colorimetric technique enabled a differentiation to be made between total normal and total uremic blood concentrations. Moreover, they proposed that this innovative sensor also makes it easier to quantify the protein-bound uremic toxins, which can greatly decrease analysis expenses.

#### 2.3.3 Other methods

The labeled immunoassay known as the enzyme-linked immunosorbent assay (ELISA) is regarded as the conventional standard technique for immunoassays [108]. This extensively utilized approach, which is one of the most common laboratory procedures in clinical, translational, and fundamental sciences as well as clinical medicine [109], allows for the precise detection of a wide range of target analytes in various types of samples. Despite its many benefits, ELISA has some drawbacks such as complicated assay procedures, and lack of sensitivity for some analytes. In addition, the main problem of ELISA is that the antibody easily detects other substances with similar epitopes, therefore false positive or negative results is possible [110].

In a recent study by Duan et al. [111], a correlation study was carried out to evaluate the relationship between renal function indicators and blood IS levels identified in CKD patients using a commercial ELISA kit and UPLC-MS/MS. They demonstrated that the ELISA method is equally effective as UPLC-MS/MS in quantifying serum IS levels, confirming the idea that ELISA would be a tool for quantification of serum IS levels in dialysis patients in order to stop the progression of CKD.

**Fig. 5:**
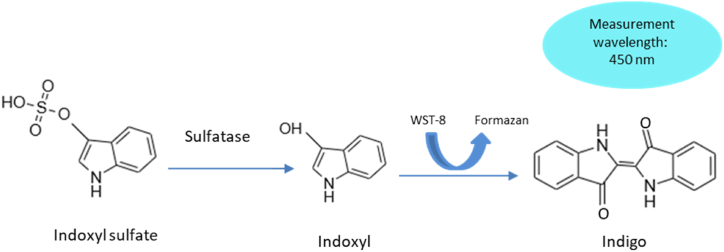
The enzymatic methodology for measuring indoxyl sulfate (IS).

In addition, Abe et al. [112] developed the “NIPRO” IS Assay Kit which includes a reagent to estimate total (free and albumin-bound) IS using enzymatic technique (Fig. 5). The WST-8 dye used in this kit, which belongs to a more recent family of formazan-based dyes [113], converted indoxyl into indigo as a colorful compound. It eliminated the need for pretreatment, such as deproteinization, and employed serum and plasma as samples. It was claimed that with the aid of this novel reagent, numerous samples can be analyzed rapidly and simply using an automatic biochemical analyzer. For evaluating the plasma of dialysis patients, the IS Assay Kit “NIPRO” demonstrated adequate performance and exhibited a good agreement with the traditional HPLC approach.

### 2.4 Future development

Technological advancements in instrument sensitivity and precision have facilitated the identification of metabolites such as IS with significantly higher confidence. The development of analytical methods is centered on the adoption of environmentally friendly and sustainable methodologies about sample preparation techniques. Additionally, an important advancement that can minimize systematic errors and progress the analytical workflow is the development of automated, miniature sample preparation techniques with green chemical properties in developing analytical methods. In this regard, applying deep eutectic solvents (DESs) has received a lot of interest, lately [114,115]. With an emphasis on the benefits of DES-based techniques over conventional ones, particular attention has been paid to the practical applications for different biological matrix such as plasma, urine and etc. Moreover, developing new analytical techniques based on nanoparticles is a new field for analysis of biomarkers [116]. They could be applied for the quantification of IS as simple and fast analytical methods. In addition, reviewed sample preparation methods could be applied for other protein-bound uremic toxins in biological samples to clean-up matrix samples and increase the sensitivity and repeatability of analytical techniques.

## 3 Conclusion

The ability to identify free and total forms of IS in biofluids using sensitive and selective approaches is critically important from the therapeutic perspective. In patients with CKD, the routine assessment of IS levels can contribute to predicting disease progression and determining the efficacy of treatments like dialysis or combination therapy. This review, from the analytical point of view, summarizes the available reported analytical methods for the assay of IS in different biological samples including LC, GC, electrochemical, spectroscopic, and other methods. Diverse sample preparation methods were proposed for the analysis of IS in biological samples. In developing analytical method, interferences can significantly affect accuracy, precision, and reliability. In addition, the presence of matrix interferences can cause baseline noise and reduce the signal-to-noise ratio. By removing interferences, the sensitivity of the method is enhanced, allowing for the detection of low concentrations of the analyte. Generally, sample preparation is selected based on the type of biological sample i.e., plasma or urine, and applied instrumental analysis for quantifying analytes. The sensitivity and accuracy of a certain analytical method with the same sample preparation and instrumental analytical method based on the type of biological sample could be different. Overviewing the informative data present in the established methods allow researchers better understand the robustness of the current techniques in measuring IS in biological samples.

## Funding

The current study results from Samira Shafiee's Ph.D. thesis. Financial support was received from 10.13039/501100004366Tabriz University of Medical Sciences, Iran, under grant number (71428).

## Availability of data and materials

The data used to support the findings of this study are included within the article.

## CRediT authorship contribution statement

**Samira Shafiee:** Writing – original draft, Investigation. **Siavoush Dastmalchi:** Writing – review & editing, Supervision. **Afshin Gharekhani:** Writing – review & editing, Conceptualization. **Ali Shayanfar:** Writing – review & editing, Supervision, Conceptualization.

## Declaration of competing interest

The authors declare that they have no known competing financial interests or personal relationships that could have appeared to influence the work reported in this paper.

## References

[1] Pizzorno J. (2015). The kidney dysfunction epidemic, part 1: causes. Integr Clin Med.

[2] Vanholder R.C., Eloot S., Glorieux G.L. (2016). Future avenues to decrease uremic toxin concentration. Am. J. Kidney Dis..

[3] Fernandes S.R., Meireles A.N., Marques S.S., Silva L., Barreiros L., Sampaio-Maia B., Miró M., Segundo M.A. (2022). Sample preparation and chromatographic methods for the determination of protein-bound uremic retention solutes in human biological samples: an overview. J. Chromatogr. B.

[4] Dobre M.A., Meyer T.W., Hostetter T.H. (2020). Chronic Renal Disease.

[5] Meijers B.K., De Loor H., Bammens B., Verbeke K., Vanrenterghem Y., Evenepoel P. (2009). p-Cresyl sulfate and indoxyl sulfate in hemodialysis patients. Clin. J. Am. Soc. Nephrol..

[6] Fujii H., Goto S., Fukagawa M. (2018). Role of uremic toxins for kidney, cardiovascular, and bone dysfunction. Toxins.

[7] Valkenburg S., Glorieux G., Vanholder R. (2021). Uremic toxins and cardiovascular system. Cardiol. Clin..

[8] Colombo G., Astori E., Landoni L., Garavaglia M.L., Altomare A., Lionetti M.C., Gagliano N., Giustarini D., Rossi R., Milzani A. (2022). Effects of the uremic toxin indoxyl sulphate on human microvascular endothelial cells. J. Appl. Toxicol..

[9] Barreto F.C., Barreto D.V., Liabeuf S., Drüeke T.B., Massy Z.A. (2009). Seminars in Dialysis.

[10] Oladi-Ghadikolaei R., Aliasgharzadeh A., Shayanfar A., Soleymani J., Moradi M., Jouyban A., Tayebi Khosroshahi H. (2023). Serum levels of indoxyl sulfate and P-cresol in type II diabetic patients with and without nephropathy. Iran J Kidney Dis.

[11] Watanabe S., Fujii H., Kono K., Watanabe K., Goto S., Sakamoto S., Nishi S. (2019). Changes in serum indoxyl sulfate levels after acute myocardial infarction and the correlation with kidney injury: an observational study. Ren Replace Ther.

[12] Lin C.-N., Wu I.-W., Huang Y.-F., Peng S.-Y., Huang Y.-C., Ning H.-C. (2019). Measuring serum total and free indoxyl sulfate and p-cresyl sulfate in chronic kidney disease using UPLC-MS/MS. J. Food Drug Anal..

[13] Lin C.J., Chen H.H., Pan C.F., Chuang C.K., Wang T.J., Sun F.J., Wu C.J. (2011). Cresylsulfate and indoxyl sulfate level at different stages of chronic kidney disease. J Clin Lab.

[14] Norouzi F., Gharekhani A., Jouyban A., Shayanfar A. (2021). Spectrofluorimetric determination of indoxyl sulfate in human plasma after salting-out assisted liquid–liquid extraction. Chem. Pap..

[15] Tan X., Cao X., Zou J., Shen B., Zhang X., Liu Z., Lv W., Teng J., Ding X. (2017). Indoxyl sulfate, a valuable biomarker in chronic kidney disease and dialysis. Hemodial. Int..

[16] Berg A.H., Kumar S., Karumanchi S.A. (2022). Indoxyl sulfate in uremia: an old idea with updated concepts. J. Clin. Invest..

[17] Gao H., Liu S. (2017). Role of uremic toxin indoxyl sulfate in the progression of cardiovascular disease. Life Sci..

[18] Lu C., Wu L., Tang M.Y., Liu Y.F., Liu L., Liu X.Y., Zhang C., Huang L. (2023). Indoxyl sulfate in atherosclerosis. Toxicol. Lett..

[19] Ershadi S., Shayanfar A. (2018). Are LOD and LOQ reliable parameters for sensitivity evaluation of spectroscopic methods?. J. AOAC Int..

[20] Shayanfar A. (2020). Beware of bar charts for plotting calibration curves for analytical method development. J. AOAC Int..

[21] Shayanfar A. (2021). A critical issue in calibration curve with logarithmic scale. ImmunoAnalysis.

[22] Ahmed S., Sparidans R.W., Lu J., Mihaila S.M., Gerritsen K.G., Masereeuw R. (2022). A robust, accurate, sensitive LC–MS/MS method to measure indoxyl sulfate, validated for plasma and kidney cells. Biomed. Chromatogr..

[23] Shu C., Chen X., Xia T., Zhang F., Gao S., Chen W. (2016). LC–MS/MS method for simultaneous determination of serum p‐cresyl sulfate and indoxyl sulfate in patients undergoing peritoneal dialysis. Biomed. Chromatogr..

[24] Baird S., Clinton Frazee C., Garg U. (2022). Clinical Applications of Mass Spectrometry in Biomolecular Analysis: Methods and Protocols.

[25] Giebułtowicz J., Korytowska N., Sankowski B., Wroczyński P. (2016). Development and validation of a LC-MS/MS method for quantitative analysis of uraemic toxins p-cresol sulphate and indoxyl sulphate in saliva. Talanta.

[26] Kanemitsu Y., Asaji K., Matsumoto Y., Tsukamoto H., Saigusa D., Mukawa C., Tachikawa T., Abe T., Tomioka Y. (2017). Simultaneous quantitative analysis of uremic toxins by LC–MS/MS with a reversed-phase/cation-exchange/anion-exchange tri-modal mixed-mode column. J. Chromatogr. B.

[27] de Loor H., Poesen R., De Leger W., Dehaen W., Augustijns P., Evenepoel P., Meijers B. (2016). A liquid chromatography–tandem mass spectrometry method to measure a selected panel of uremic retention solutes derived from endogenous and colonic microbial metabolism. Anal. Chim. Acta.

[28] Fabresse N., Uteem I., Lamy E., Massy Z., Larabi I.A., Alvarez J.-C. (2020). Quantification of free and protein bound uremic toxins in human serum by LC-MS/MS: comparison of rapid equilibrium dialysis and ultrafiltration. Clin. Chim. Acta.

[29] Zhu W., Stevens A.P., Dettmer K., Gottfried E., Hoves S., Kreutz M., Holler E., Canelas A.B., Kema I., Oefner P.J. (2011). Quantitative profiling of tryptophan metabolites in serum, urine, and cell culture supernatants by liquid chromatography–tandem mass spectrometry. Anal. Bioanal. Chem..

[30] Ragi N., Pallerla P., Babi Reddy Gari A.R., Lingampelly S.S., Ketavarapu V., Addipilli R., Chirra N., Kantevari S., Yadla M., Sripadi P. (2023). Assessment of uremic toxins in advanced chronic kidney disease patients on maintenance hemodialysis by LC-ESI-MS/MS. J Metabolomics.

[31] Itoh Y., Ezawa A., Kikuchi K., Tsuruta Y., Niwa T. (2012). Protein-bound uremic toxins in hemodialysis patients measured by liquid chromatography/tandem mass spectrometry and their effects on endothelial ROS production. Anal. Bioanal. Chem..

[32] Choi J.M., Park W.S., Song K.Y., Lee H.J., Jung B.H. (2016). Development of simultaneous analysis of tryptophan metabolites in serum and gastric juice–an investigation towards establishing a biomarker test for gastric cancer diagnosis. Biomed. Chromatogr..

[33] Monošík R., Dragsted L.O. (2016). A versatile UHPLC–MSMS method for simultaneous quantification of various alcohol intake related compounds in human urine and blood. Anal. Methods.

[34] Zhang A., Rijal K., Ng S.K., Ravid K., Chitalia V. (2017). A mass spectrometric method for quantification of tryptophan-derived uremic solutes in human serum. J Biol Methods.

[35] Wang Z., Jiang H., Chen X., Song X., Xu F., Chen F., Mao Z., Gao S., Chen W. (2020). A rapid and sensitive method for simultaneous determination of eight protein-bound uremic toxins in human serum by UHPLC-MS/MS: application in assessing peritoneal dialysis. J. Pharm. Biomed. Anal..

[36] Prokopienko A.J., West R.E., Stubbs J.R., Nolin T.D. (2019). Development and validation of a UHPLC-MS/MS method for measurement of a gut-derived uremic toxin panel in human serum: an application in patients with kidney disease. J. Pharm. Biomed. Anal..

[37] Oda A., Suzuki Y., Sato B., Sato H., Tanaka R., Ono H., Ando T., Shin T., Mimata H., Itoh H. (2022). Highly sensitive simultaneous quantification of indoxyl sulfate and 3‐carboxy‐4‐methyl‐5‐propyl‐2‐furanpropanoic acid in human plasma using ultra‐high‐performance liquid chromatography coupled with tandem mass spectrometry. J. Separ. Sci..

[38] Boelaert J., Lynen F., Glorieux G., Eloot S., Van Landschoot M., Waterloos M.-A., Sandra P., Vanholder R. (2013). A novel UPLC–MS–MS method for simultaneous determination of seven uremic retention toxins with cardiovascular relevance in chronic kidney disease patients. Anal. Bioanal. Chem..

[39] Olesova D., Galba J., Piestansky J., Celusakova H., Repiska G., Babinska K., Ostatnikova D., Katina S., Kovac A. (2020). A novel UHPLC-MS method targeting urinary metabolomic markers for autism spectrum disorder. Metabolites.

[40] Zhang Y., Gu L., Jiang Y., Bi K., Chen X. (2016). Quantitative analysis of biomarkers of liver and kidney injury in serum and urine using ultra‐fast liquid chromatography with tandem mass spectrometry coupled with a hydrophilic interaction chromatography column: application to monitor injury induced by Euphorbiae pekinensis Radix. J. Separ. Sci..

[41] Silva L.A., Campagnolo S., Fernandes S.R., Marques S.S., Barreiros L., Sampaio-Maia B., Segundo M.A. (2023). Rapid and sustainable HPLC method for the determination of uremic toxins in human plasma samples. Anal. Bioanal. Chem..

[42] Al Za'abi M., Ali B., Al Toubi M. (2013). HPLC–fluorescence method for measurement of the uremic toxin indoxyl sulfate in plasma. J. Chromatogr. Sci..

[43] Calaf R., Cerini C., Génovésio C., Verhaeghe P., Jourde-Chiche N., Bergé-Lefranc D., Gondouin B., Dou L., Morange S., Argilés A. (2011). Determination of uremic solutes in biological fluids of chronic kidney disease patients by HPLC assay. J. Chromatogr. B.

[44] de Loor H., Meijers B.K., Meyer T.W., Bammens B., Verbeke K., Dehaen W., Evenepoel P. (2009). Sodium octanoate to reverse indoxyl sulfate and p-cresyl sulfate albumin binding in uremic and normal serum during sample preparation followed by fluorescence liquid chromatography. J. Chromatogr. A.

[45] Zou C., Lu F., Wu Y., Lin Q., Liu X. (2012). Indoxyl sulfate serum level in chronic renal failure patients detected using fluorescence-HPLC. Kidney Res Clin Pract.

[46] Wu C., Shi C.-g., Li M., Pan B.-y., Xing X.-m., Feng Z.-q., Xie Z.-y. (2010). Determination of indoxyl sulfate in human serum by HPLC-FLU and its application in hemodialysis patients. Chin. J. Pharmacol. Toxicol..

[47] Pretorius C.J., McWhinney B.C., Sipinkoski B., Johnson L.A., Rossi M., Campbell K.L., Ungerer J.P. (2013). Reference ranges and biological variation of free and total serum indoxyl-and p-cresyl sulphate measured with a rapid UPLC fluorescence detection method. Clin. Chim. Acta.

[48] Valko-Rokytovská M., Očenáš P., Salayová A., Kostecká Z. (2018). New developed UHPLC method for selected urine metabolites. J. Chromatogr. Separ. Tech..

[49] Stanfel L.A., Gulyassy P.F., Jarrard E.A. (1986). Determination of indoxyl sulfate in plasma of patients with renal failure by use of ion-pairing liquid chromatography. Clin. Chem..

[50] Alothman Z.A., Alanazi A.G., Ali I. (2020). A comparative and simultaneous analysis of indoxyl sulfate and sodium butyrate in human plasma by SPE and HPLC methods for kidney patients. J. Chromatogr. B Biomed. Appl..

[51] Wang C.-H., Lai Y.-H., Kuo C.-H., Lin Y.-L., Tsai J.-P., Hsu B.-G. (2019). Association between serum indoxyl sulfate levels and endothelial function in non-dialysis chronic kidney disease. Toxins.

[52] Koike H., Morita T., Tatebe J., Watanabe I., Koike M., Yao S., Shinohara M., Yuzawa H., Suzuki T., Fujino T. (2019). The relationship between serum indoxyl sulfate and the renal function after catheter ablation of atrial fibrillation in patients with mild renal dysfunction. Heart Ves..

[53] Li Q., Zhang S., Wu Q.-J., Xiao J., Wang Z.-H., Mu X.-W., Zhang Y., Wang X.-N., You L.-L., Wang S.-N. (2022). Serum total indoxyl sulfate levels and all-cause and cardiovascular mortality in maintenance hemodialysis patients: a prospective cohort study. BMC Nephrol..

[54] Lin T.-J., Hsu B.-G., Wang J.-H., Lai Y.-H., Dongoran R.A., Liu C.-H. (2020). Serum indoxyl sulfate as a potential biomarker of aortic arterial stiffness in coronary artery disease. Nutr. Metab..

[55] Yoshida T., Tsujimoto M., Kawakami S., Fujioka H., Irie Y., Nakatani S., Iso A., Sugiyama A., Miyake M., Hirato K. (2022). Research on the relationship between serum indoxyl sulfate concentration and iron dynamics index in patients with end-stage kidney disease: a cross-sectional study. Ren Replace Ther.

[56] Barreto F.C., Barreto D.V., Liabeuf S., Meert N., Glorieux G., Temmar M., Choukroun G., Vanholder R., Massy Z.A. (2009). Serum indoxyl sulfate is associated with vascular disease and mortality in chronic kidney disease patients. Eutox.

[57] Bolati D., Shimizu H., Yisireyili M., Nishijima F., Niwa T. (2013). Indoxyl sulfate, a uremic toxin, downregulates renal expression of Nrf2 through activation of NF-κB. BMC Nephrol..

[58] Fan P.-C., Chang J.C.-H., Lin C.-N., Lee C.-C., Chen Y.-T., Chu P.-H., Kou G., Lu Y.-A., Yang C.-W., Chen Y.-C. (2019). Serum indoxyl sulfate predicts adverse cardiovascular events in patients with chronic kidney disease. J. Formos. Med. Assoc..

[59] Lin C.-J., Liu H.-L., Pan C.-F., Chuang C.-K., Jayakumar T., Wang T.-J., Chen H.-H., Wu C.-J. (2012). Indoxyl sulfate predicts cardiovascular disease and renal function deterioration in advanced chronic kidney disease. Arch. Med. Res..

[60] Takkavatakarn K., Phannajit J., Udomkarnjananun S., Tangchitthavorngul S., Chariyavilaskul P., Sitticharoenchai P., Praditpornsilpa K., Eiam-Ong S., Susantitaphong P. (2022). Association between indoxyl sulfate and dialysis initiation and cardiac outcomes in chronic kidney disease patients. Int. J. Nephrol. Renovascular Dis..

[61] Adesso S., Popolo A., Bianco G., Sorrentino R., Pinto A., Autore G., Marzocco S. (2013). The uremic toxin indoxyl sulphate enhances macrophage response to LPS. PLoS One.

[62] Barreto F.C., Barreto D.V., Canziani M.E.F., Tomiyama C., Higa A., Mozar A., Glorieux G., Vanholder R., Massy Z., Carvalho A.B.d. (2014). Association between indoxyl sulfate and bone histomorphometry in pre-dialysis chronic kidney disease patients. J Bras Nefrol.

[63] Wu C.J., Chen C.Y., Lai T.S., Wu P.C., Chuang C.K., Sun F.J., Liu H.L., Chen H.H., Yeh H.I., Lin C.S., Lin C.J. (2019). Correction: the role of indoxyl sulfate in renal anemia in patients with chronic kidney disease. Oncotarget.

[64] Chiang C.K., Tanaka T., Inagi R., Fujita T., Nangaku M. (2011). Indoxyl sulfate, a representative uremic toxin, suppresses erythropoietin production in a HIF-dependent manner. Lab. Invest..

[65] Hamza E., Vallejo-Mudarra M., Ouled-Haddou H., García-Caballero C., Guerrero-Hue M., Santier L., Rayego-Mateos S., Larabi I.A., Alvarez J.C., Garçon L., Massy Z.A., Choukroun G., Moreno J.A., Metzinger L., Meuth V.M. (2023). Indoxyl sulfate impairs erythropoiesis at BFU-E stage in chronic kidney disease. Cell. Signal..

[66] Hsu C.-C., Lu Y.-C., Chiu C.-A., Yu T.-H., Hung W.-C., Wang C.-P., Lu L.-F., Chung F.-M., Lee Y.-J., Tsai I.-T. (2013). Levels of indoxyl sulfate are associated with severity of coronary atherosclerosis. Clin. Invest. Med..

[67] Taki K., Tsuruta Y., Niwa T. (2007). Indoxyl sulfate and atherosclerotic risk factors in hemodialysis patients. Am. J. Nephrol..

[68] Lin C.-J., Pan C.-F., Chuang C.-K., Liu H.-L., Sun F.-J., Wang T.-J., Chen H.-H., Wu C.-J. (2014). Association of indoxyl sulfate with fibroblast growth factor 23 in patients with advanced chronic kidney disease. Am. J. Med. Sci..

[69] Holle J., Querfeld U., Kirchner M., Anninos A., Okun J., Thurn-Valsassina D., Bayazit A., Niemirska A., Canpolat N., Bulut I.K. (2019). Indoxyl sulfate associates with cardiovascular phenotype in children with chronic kidney disease. Pediatr. Nephrol..

[70] Caldiroli L., Armelloni S., Eskander A., Messa P., Rizzo V., Margiotta E., Cesari M., Vettoretti S. (2021). Association between the uremic toxins indoxyl-sulfate and p-cresyl-sulfate with sarcopenia and malnutrition in elderly patients with advanced chronic kidney disease. Exp. Gerontol..

[71] Goto S., Fujii H., Hamada Y., Yoshiya K., Fukagawa M. (2010). Association between indoxyl sulfate and skeletal resistance in hemodialysis patients. Ther. Apher. Dial..

[72] Wu C.-C., Hsieh M.-Y., Hung S.-C., Kuo K.-L., Tsai T.-H., Lai C.-L., Chen J.-W., Lin S.-J., Huang P.-H., Tarng D.-C. (2016). Serum indoxyl sulfate associates with postangioplasty thrombosis of dialysis grafts. J. Am. Soc. Nephrol..

[73] Wang S.-C., Lai Y.-H., Liu C.-H., Wang C.-H., Hsu B.-G., Tsai J.-P. (2021). Association between serum indoxyl sulfate levels with carotid-femoral pulse wave velocity in patients with chronic kidney disease. Ren. Fail..

[74] Caggiano G., Amodio L., Stasi A., Colabufo N.A., Colangiulo S., Pesce F., Gesualdo L. (2023). Gut-derived uremic toxins in CKD: an improved approach for the evaluation of serum indoxyl sulfate in clinical practice. Int. J. Mol. Sci..

[75] Summers S., Quimby J.M., Phillips R.K., Stockman J., Isaiah A., Lidbury J.A., Steiner J.M., Suchodolski J. (2020). Preliminary evaluation of fecal fatty acid concentrations in cats with chronic kidney disease and correlation with indoxyl sulfate and p‐cresol sulfate. J. Vet. Intern. Med..

[76] Korytowska N., Wyczałkowska-Tomasik A., Paczek L., Giebułtowicz J. (2021). Evaluation of salivary indoxyl sulfate with proteinuria for predicting graft deterioration in kidney transplant recipients. Toxins.

[77] Farowski F., Els G., Tsakmaklis A., Higgins P.G., Kahlert C.R., Stein-Thoeringer C.K., Bobardt J.S., Dettmer-Wilde K., Oefner P.J., Vehreschild J.J. (2019). Assessment of urinary 3-indoxyl sulfate as a marker for gut microbiota diversity and abundance of Clostridiales. Gut Microb..

[78] Ma Y.-r., Xin M.-y., Li K., Wang H., Rao Z., Liu T.-x., Wu X.-a. (2020). An LC-MS/MS analytical method for the determination of uremic toxins in patients with end-stage renal disease. J. Pharm. Biomed. Anal..

[79] Zeng Y., Luo L., Hou W., Lu B., Gong J., Chen J., Zhang X., Han B., Xie Z., Liao Q. (2017). Targeted metabolomics analysis of aromatic amino acids and their gut microbiota–host cometabolites in rat serum and urine by liquid chromatography coupled with tandem mass spectrometry. J. Separ. Sci..

[80] Rankin-Turner S., Heaney L.M. (2022). Mass spectrometry in the clinical laboratory. A short journey through the contribution to the scientific literature by CCLM. Clin. Chem. Lab. Med..

[81] Zhou X., Zhang W., Ouyang Z. (2022). Recent advances in on-site mass spectrometry analysis for clinical applications. TrAC - Trends Anal Chem.

[82] Vaghela A., Patel A., Patel A., Vyas A., Patel N. (2016). Sample preparation in bioanalysis: a review. Int J Sci Technol Res.

[83] Mathew J., Sankar P., Varacallo M. (2023). StatPearls [Internet].

[84] Sarigul N., Korkmaz F., Kurultak İ. (2019). A new artificial urine protocol to better imitate human urine. Sci. Rep..

[85] Soylak M., Jagirani M.S. (2021). Extraction techniques used for the removal of pharmaceuticals from environmental samples. Pharm. Sci..

[86] Castro-Perez J., Prakash C. (2020). Recent advances in mass spectrometric and other analytical techniques for the identification of drug metabolites, Identification Quantification of Drugs. Metabolites, Drug Metabolizing Enzymes, Transporters.

[87] Niu Z., Zhang W., Yu C., Zhang J., Wen Y. (2018). Recent advances in biological sample preparation methods coupled with chromatography, spectrometry and electrochemistry analysis techniques. TrAC - Trends Anal Chem.

[88] Wang G., Korfmacher W.A. (2009). Development of a biomarker assay for 3‐indoxyl sulfate in mouse plasma and brain by liquid chromatography/tandem mass spectrometry. Rapid Commun. Mass Spectrom..

[89] Kong R., Ahuja S., Dong M.W. (2005).

[90] Queiroz M.E.C., de Souza I. (2018). Sample preparation techniques for biological samples. J. Chromatogr. Sci..

[91] Cheng F., Hsieh M., Chou C., Hsu W., Lee Y. (2015). Detection of indoxyl sulfate levels in dogs and cats suffering from naturally occurring kidney diseases. Vet J VET J.

[92] Paats J., Adoberg A., Arund J., Dhondt A., Fernström A., Fridolin I., Glorieux G., Leis L., Luman M., Gonzalez-Parra E. (2020). Serum levels and removal by haemodialysis and haemodiafiltration of tryptophan-derived uremic toxins in ESKD patients. Int. J. Mol. Sci..

[93] Rockwood A.L., Kushnir M.M., Clarke N.J. (2018). Mass spectrometry. Clin Mass Spectrom.

[94] Niwa T. (2011). Update of uremic toxin research by mass spectrometry. Mass Spectrom. Rev..

[95] Sparkman O.D., Penton Z., Kitson F.G. (2011).

[96] McEwen C.N., McKay R.G. (2005). A combination atmospheric pressure LC/MS:GC/MS ion source: advantages of dual AP-LC/MS:GC/MS instrumentation. J. Am. Soc. Mass Spectrom..

[97] Omori K., Katakami N., Arakawa S., Yamamoto Y., Ninomiya H., Takahara M., Matsuoka T.-a., Tsugawa H., Furuno M., Bamba T. (2020). Identification of plasma inositol and Indoxyl sulfate as novel biomarker candidates for atherosclerosis in patients with type 2 diabetes.-findings from Metabolome analysis using GC/MS. J. Atherosclerosis Thromb..

[98] Elsonbaty A., Abdel-Raoof A.M., Abdulwahab S., Hassan W.S., Eissa M.S. (2021). Electrochemical determination of amprolium hydrochloride in chicken meats and eggs: food safety control and theoretical study. J. Electrochem. Soc..

[99] Elsonbaty A., Attala K. (2021). Application of experimental design approaches and in silico molecular docking on the host-guest complexes with cyclodextrin for the analysis of benazepril hydrochloride in pharmaceutical formulation. J. Electrochem. Soc..

[100] Mostafa A.E., Elsonbaty A., Attala K., Abdelshakour M.A., Abdel Salam R.A., Hadad G.M., Eissa M.S. (2023). Miniaturized chip integrated ecological sensor for the quantitation of milnacipran hydrochloride in the presence of its impurities in dosage form and human plasma. J. Electrochem. Soc..

[101] Otaif K.D., Fouad M.M., Rashed N.S., Hosni N.Y., Elsonbaty A., Elgazzar E. (2023). Green Prospective approach of chromium zinc oxide nanoparticles for highly ultrasensitive electrochemical detection of anti-hypotensive medication in various matrices. ACS Omega.

[102] Abdel-Raoof A.M., Fouad M.M., Rashed N.S., Hosni N.Y., Elsonbaty A., Abdel-Fattah A. (2023). Potentiometric determination of mebeverine hydrochloride antispasmodic drug based on molecular docking with different ionophores host–guest inclusion as a theoretical study. RSC Adv..

[103] Fujita K., Nonaka T., Kutsuno R., Ichida K. (2022). Electrochemical sensing of the secretion of indoxyl sulfate in a rat intestinal loop using a self-assembled monolayer-modified gold bead electrode. Talanta.

[104] Filik H., A Avan A., Aydar S. (2016). Voltammetric sensing of uremic toxin indoxyl sulfate using high performance disposable screen-printed graphene electrode. Curr. Pharm. Anal..

[105] Holmar J., Arund J., Uhlin F., Tanner R., Fridolin I. (2013). World Congress on Medical Physics and Biomedical Engineering May 26-31, 2012.

[106] Rajasekaran R., Aruna P.R., Koteeswaran D., Bharanidharan G., Baludavid M., Ganesan S. (2014). Steady-state and time-resolved fluorescence spectroscopic characterization of urine of healthy subjects and cervical cancer patients. J. Biomed. Opt..

[107] Schiefer E.M., Santos A.F., Muller M., Stinghen A.E., Negri L.H., Fabris J.L. (2021). Protein-bound uremic toxins quantification by a colorimetric sensor based on the oxidation of silver nanoparticles. IEEE Sensor. J..

[108] Alhajj M., Farhana A. (2023).

[109] Drijvers J.M., Awan I.M., Perugino C.A., Rosenberg I.M., Pillai S. (2017). Basic Science Methods for Clinical Researchers.

[110] Hosseini S., Vázquez-Villegas P., Rito-Palomares M., Martinez-Chapa S.O. (2018). Enzyme-linked Immunosorbent Assay (ELISA). SpringerBriefs in Applied Sciences and Technology.

[111] Duan S., Pi J., Wang C.-H., Hou Y.-C., Lee C.-Y.A., Lin C.-J., Shi L., Young K.-C., Sun H.-Y. (2022). Assessment of ELISA-based method for the routine examination of serum indoxyl sulfate in patients with chronic kidney disease. Heliyon.

[112] Abe T., Onoda M., Matsuura T., Sugimura J., Obara W., Sasaki N., Kato T., Tatsumi K., Maruyama T. (2021). Evaluation of a new measurement method of indoxyl sulfate in hemodialysis patients. Ther. Apher. Dial..

[113] Stoddart M.J. (2011). Stoddart, Protocols, WST-8 analysis of cell viability during osteogenesis of human mesenchymal stem cells. Methods Mol. Biol..

[114] Grecco C.F., de Souza I.D., Queiroz M.E.C. (2021). Novel materials as capillary coatings for in-tube solid-phase microextraction for bioanalysis. J. Separ. Sci..

[115] Soltanmohammadi F., Jouyban A., Shayanfar A. (2021). New aspects of deep eutectic solvents: extraction, pharmaceutical applications, as catalyst and gas capture. Chem. Pap..

[116] Mohammadzadeh Abachi S., Rezaei H., Khoubnasabjafari M., Jouyban-Gharamaleki V., Rahimpour E., Jouyban A. (2023). Utilizing nanoparticle catalyzed TMB/H2O2 system for determination of aspirin in exhaled breath condensate. Pharm. Sci..

